# Traditional Herbal Medicine Mediated Regulations during Head and Neck Carcinogenesis

**DOI:** 10.3390/biom10091321

**Published:** 2020-09-15

**Authors:** Xiang-Yun Lan, Tzu-Ting Chung, Chien-Ling Huang, Yi-Jang Lee, Wan-Chun Li

**Affiliations:** 1 Institute of Oral Biology, School of Dentistry, National Yang-Ming University, Taipei 11221, Taiwan; xiangyun1002@gmail.com; 2 Department of Dentistry, School of Dentistry, National Yang-Ming University, Taipei 11221, Taiwan; chungtzuting@gm.ym.edu.tw; 3 Department of Health Technology and Informatics (HTI), The Hong Kong Polytechnic University (PolyU), Hung Hom, Kowloon, Hong Kong, SAR, China; cl.huang@polyu.edu.hk; 4 Department of Biomedical Imaging and Radiological Sciences, National Yang-Ming University, Taipei 11221, Taiwan; yjlee2@ym.edu.tw; 5 Cancer Progression Research Center, National Yang-Ming University, Taipei 11221, Taiwan

**Keywords:** head and neck cancer, traditional herbal medicine, tumor growth, metastasis, angiogenesis, xerostomia, oral mucositis, integrative therapy

## Abstract

Head and neck squamous cell carcinoma (HNSCC) is one of the most prevalent neoplasms worldwide. It is well recognized that environmental challenges such as smoking, viral infection and alcohol consumption are key factors underlying HNSCC pathogenesis. Other than major clinical interventions (e.g., surgical resection, chemical and radiotherapy) that have been routinely practiced over years, adjuvant anticancer agents from Traditional Herbal Medicine (THM) are proposed, either alone or together with conventional therapies, to be experimentally effective for improving treatment efficacy in different cancers including HNSCCs. At a cellular and molecular basis, THM extracts could modulate different malignant indices via distinct signaling pathways and provide better control in HNSCC malignancy and its clinical complications such as radiotherapy-induced xerostomia/oral mucositis. In this article, we aim to systemically review the impacts of THM in regulating HNSCC tumorous identities and its potential perspective for clinical use.

## 1. Introduction

Head and Neck Squamous Cell carcinomas (HNSCCs) influence a variety of anatomic sites, including the oral cavity, oropharynx, nasopharynx, hypopharynx, larynx, and salivary glands [1]. Different stimuli including smoking, alcohol consumption, viral infection and imbalanced metabolism serve as potential triggers for HNSCC development [2,3,4]. Despite advances in surgical technology and development of adjuvant treatments, the 5-year survival rate of HNSCC remains low (32% to 53%), mainly resulting from frequent local invasion, regional lymph node metastasis and overgrowth of drug-resistant HNSCC cells in response to conventional treatments [2,5]. More recently, the concept of “Integrative medicine” has gained attention. This concept focuses on a collaborative application to HNSCC patient care that involves a strategy to bring conventional and complementary approaches together in a coordinated way [6]. Among different integrative therapies, Traditional Herbal Medicine (THM) might be the most common biologically-based therapy used by patients with HNSCCs [7]. While scientific evidence is fairly limited regarding the efficacy of THM compounds for cancer prevention and treatment, abundant data supporting integrative therapies for symptom management in clinic were defined by randomized controlled trials. A systematic review suggested that around 6–79% of HNSCC patients routinely receive integrative therapy, depending on the methodology of data collection [7,8]. Collectively, not only in Asian countries, global recognition for THM compounds for treating deleterious physiological abnormalities such as HNSCCs is drawing more attention, making it essential to provide better understanding, from a scientific point of view, of the working mechanisms of THM in regulating oncogenicity. This review article therefore seeks to elucidate the cellular and molecular basis of THM-mediated anticancer impacts for different cancerous identity including cell growth/survival, cell migration/metastasis, angiogenesis, therapeutic sensitivity and radiotherapy-induced complications in HNSCCs. Despite different disease etiology, in order to increase the sizes of reference literature, in addition to oral, tongue, buccal, laryngeal and hypopharyngeal cancers, we also recruited esophageal and nasopharyngeal carcinoma, which are anatomically similar to HNSCCs.

## 2. Multifaceted Regulations by THM in HNSCCs

### 2.1. THM Extracts Lessen Cell Growth/Survival in HNSCCs

Suppression of tumor cell growth/survival is the most convincing strategy to command cancer oncogenesis. Numerous studies were previously reported showing that different THM compounds could both directly regulate the survival and proliferation of cancers and enhance the sensitivity of tumor cells to clinical interventions [9,10,11]. Different THM compounds including curcumin, triptolide, cucurbitacins, oridonin, artesunate, β-elemene and cepharanthine could all influence cell cycle-related molecules such as cyclins and caspases, thus leading to cell cycle arrest and promoting cell apoptosis in HNSCC cells [12,13,14,15,16,17,18,19,20,21]. At the molecular level, THM compounds seem to regulate tumorous properties through modulation of different external and intrinsic apoptotic signaling pathways. For instance, berberine, demethoxycurcumin, ursolic acid, tanshinones, oridonin, moscatilin and wogonin were all capable of suppressing oncogenic PI3K/Akt/mTOR and MAPK/JNK/p38 pathways thereby resulting in cell proliferation inhibition in HNSCC as well as in nasopharyngeal carcinoma [13,22,23,24,25,26,27,28]. Moreover, mitochondrial-associated apoptotic regulators BCL-2 and Bax were also important mediators for THM-related antigrowth/survival activity. While mitochondria also play an important role in cellular metabolism, it suggested that THM compounds might also be essential to elicit anti-HNSCC activity via metabolic regulation. Previous studies indicated that curcumin, oridonin, artesunate and β-elemene modulated HNSCC cell viability by affecting the BCL-2/Bax protein level [12,13,19,29,30]. Interestingly, a very recent study reporting that chrysophanol could up-regulate Reactive Oxygen Species (ROS) levels thereby regulating cell death further supports that THM compounds are likely metabolic regulating agents [31]. Another common underlying THM-mediated regulatory cue for HNSCC growth/survival is autophagy, as Epigallocatechin gallate (EGCG), dihydroartemisinin, tanshinones and wogonin were all autophagy inducers in HNSCC cells [25,32,33,34]. The experimental evidence of utilizing THM compounds as effectors for HNSCC growth/survival is summarized in Table 1.

### 2.2. THM Extracts Control Cell Motility and Angiogenesis in HNSCCs

Over the past decade, numerous studies revealed the potential that THM compounds could inhibit HNSCC metastasis and angiogenesis (Table 2). With regard to the regulation of cell motility, it is well accepted that tumor cells need to make room for movement. Metalloproteinases (MMPs) are a family of proteinases that could catalyze various components of the Extracellular Matrix (ECM). MMPs could be categorized into several subfamilies according to their substrate including collagenases, gelatinases, stromelysins, matrilysins, membrane-type MMPs (MT-MMPs) etc. Among them, gelatinases, MMP-2 and MMP-9, are frequently enriched in HNSCCs and often relate to an increased risk of metastasis [43], making MMPs a potential underlying regulator of THM-mediated migration/metastasis in HNSCCs. Indeed, it is reported that most investigations found that THM compounds could suppress different types of MMPs, mainly MMP-2 and MMP-9, suggesting that MMPs could be key molecular cues for HNSCC cell motility. At the cellular level, MMP-2 and MMP-9 were thought to affect cancer migration and invasion through their catalytic ability to degrade type IV collagen and denatured collagen (gelatin), thus weakening the structure of ECM. Interestingly, recent studies showed that MMP-2 and MMP-9 could also regulate cytokines, growth factors, chemokines, and other bioactive molecules in the process of ECM degradation, indicating that MMP-2 and MMP-9 could possibly act indirectly to control cell migration and angiogenesis [43]. In addition to different MMPs, ECM degradation for cell movement could also be controlled by inhibitory signals such as tissue inhibitors of metalloproteinases (TIMPs), endogenous inhibitors of MMPs. TIMPs exhibit fairly high affinity to MMPs by noncovalent binding, thereby blocking the active site of MMPs resulting in inactive MMPs [44]. Unexpectedly, TIMPs also contribute to the activation of MMPs. For example, the formation of MT1-MMP/TIMP-2/pro-MMP2 tri-molecule complex is required to activate conversion of pro-MMP-2 to active MMP-2 [43]; on the other hand, a higher level of TIMP-2 may saturate MT1-MMPs, thus preventing the cleavage of pro-MMP-2 [43]. In short, an optimal level of TIMPs is needed to reach the maximum activity of MMPs.

In addition to TIMPs, interleukins, interferons, epidermal growth factor (EGF), keratinocyte growth factor (KGF), nerve growth factor (NGF), vascular endothelial growth factor (VEGF), platelet-derived growth factor (PDGF), tumor necrosis factor-α (TNF-α) and transforming growth factor (TGF-β) could all transactivate MMP gene expression [78]. Other than extracellular signaling molecules, intracellular factors such as activator protein-1 (AP-1) also play an important role in regulating MMP genes. AP-1 is a transcription factor that consists of four subfamilies, Jun, Fos, Maf, and activating transcription factor (ATF) [79]. The dimeric AP-1 complex is composed of Jun and Fos proteins, all of which could bind to proximal promoter regions of different MMPs including MMP-1, -3, -7, -9, -10, -12, and -13 and thereby promote MMP mRNA levels [78]. The upstream triggers for AP-1 activity have also been studied while mitogen-activated protein kinases (MAPKs) turned out to be one of the key contributors for AP-1 activation. MAPKs consist of mitogen-activated intracellular signal-regulated kinase 1/2 (ERK1/2), stress-activated Jun N-terminal kinase (JNK), p38 kinase, and ERK5 [80]. For example, ERKs could phosphorylate ternary complex factors (TCFs), Fra1/2, and monocyte-specific enhancer binding factor 2c (MEF2C), which promote fos transcription, c-Jun activity and c-Jun expression, respectively [79]. On the other hand, JNK can directly phosphorylate c-Jun or phosphorylate ATF2 resulting in c-jun transcriptional activation. Moreover, p38 could also directly phosphorylate ATF2, MEF2C, and TCFs facilitating AP-1 transcription [79]. Lastly, Urokinase-plasminogen activator (u-PA) is also found in several studies involving ECM degradation by converting plasminogen to active plasmin which leads to MMP activation [43,81].

In addition to MMPs, epithelial-mesenchymal transition (EMT) also offers a perspective on the subject. To date, EMT is still a hypothetical phenomenon that is recognized mostly in metastatic tumor cells. EMT activation is defined as a transformation of highly differentiated, polarized, and organized epithelial cells into undifferentiated, isolated, and mesenchymal-like cells. These mesenchymal-like cells could develop anoikis resistance, have poorer cell-to-cell/cell-to-matrix adhesion, and might be more invasive and migratory [82]. Cells that undergo EMT could survive and avoid apoptosis despite the absence of ECM; in other words, mesenchymal cells could acquire anoikis resistance. At the molecular level, these cells possess higher expression of antiapoptotic genes (Bcl-2 family) and/or more active prosurvival cues (i.e., PI3K/Akt signaling pathway). Additionally, proapoptotic proteins such as p53-effector related to pmp22 (PERP), p21, Bim, Bax, and Noxa are down-regulated. The expression of mesenchymal markers such as vimentin, fibronectin, α-smooth muscle actin (SMA) as well as MMPs is also increased. Apart from anoikis resistance, EMT lets epithelial cells remodel the cytoskeleton to form invasive protrusions known as invadopodia, thus increasing the motility of the cells [82]. Also, the cell-to-cell linkage is reduced due to the loss of E-cadherin and catenin through epigenetic modifications. In contrast, N-cadherin, a mesenchymal cadherin expressed in stromal cells, is increased, thereby enhancing the ability of tumor cells to invade into the stroma [82]. Several important transcription factors such as Snail, ZEB1/2, Twist, NF-kB, and HIF1/2 are involved in EMT in a coordinative manner with other signaling pathways [82,83].

To support tumor growth, angiogenesis is also important for metastatic cancer cells while blood vessels are essential for nutrient transportation. Angiogenesis represents an outcome of balance between proangiogenic factors (VEGF, PDGR, FGF, Angiopoietin) and antiangiogenic factors (endostatin, angiostatin, and thrombospondin) [81]. In all studies we reviewed, vascular endothelial-derived growth factor (VEGF) is most frequently discussed. VEGF is an important proangiogenic and provasculogenic factor. As VEGF binds to VEGF receptors on tumor cells, downstream signaling pathways could be further activated, thus leading to greater cell proliferation, migration, survival, and better vascular permeability. Interestingly, it was found that Notch signaling is involved and promotes the formation of tip cells and filopodia, which are important for angiogenic sprouting [84]. Besides VEGF, MMPs also regulate angiogenesis in a more complicated manner. Many members of the MMP family can activate proangiogenic factors and antiangiogenic factors. For instance, MMP-9 is capable of activating VEGF while other MMPs exhibited the ability to induce basic fibroblast growth factor (bFGF), Heparin-binding EGF-like growth factor (HB-EGF), or transforming growth factor-β (TGF-β) to promote angiogenesis [85]. On the contrary, some MMPs might activate antiangiogenic factors via activation of angiostatin and endostatin, endogenous inhibitors of angiogenesis [85]. In summary, THM compounds are effective in inhibiting metastasis and angiogenesis. The possible mechanism might include the down-regulation of MMPs and EMT. THM compounds have also shown the ability to decrease VEGF levels, thus suppressing angiogenesis.

### 2.3. THM Extracts Modulate Therapeutic Sensitivity in HNSCCs

To date, the 5-year survival rates for HNSCC patients remained low once tumors metastasize to distant organs, resulting in treatment resistance to conventional therapies [86]. General clinical therapeutic schemes for HNSCC patients have been widely practiced, both with single or combinational regimes. Surgical resection, chemotherapy and radiotherapy are common choices depending on tumor sizes, locations, tumor grades and clinic stages [87,88]. Other alternative treatments such as cryotherapy [89] and photodynamic therapy (PDT) [90,91] were also tested for their efficacy for treating oral neoplasms. Targeting HNSCC-specific molecules further developed a number of targeted therapy agents including epidermal growth factor receptor (EGFR) monoclonal antibodies (cetuximab, panitumumab, zalutumumab and nimotuzumab), EGFR tyrosine kinase inhibitors (TKIs) (gefitinib, erlotinib, lapatinib, afatinib and dacomitinib) as well as vascular endothelial growth factor (VEGF) inhibitors (bevacizumab) or vascular endothelial growth factor receptor (VEGFR) inhibitors (sorafenib, sunitinib and vandetanib) [92]. With significant improvement to local control early-stage disease, multidisciplinary therapy is often applied in order to enhance prognosis for advanced HNSCCs. While the use of combinations of different therapeutic agents such as cisplatin (CDDP) +5-fluorouracil (5-FU), CDDP + radiotherapy, cetuximab + radiation (for local advanced HNSCCs) and cetuximab + chemotherapeutic drugs (for recurrent/metastatic HNSCCs) were also frequently practiced in clinic [93,94]. CDDP-based treatment, however, showed certain limitations as patients often acquired drug resistance with prolonged treatment [95,96]. At the cellular and molecular level, multiple drug resistance (MDR) could be achieved by several mechanisms: (i) modulation of drug influx/efflux capacity; (ii) increase of drug metabolism; (iii) promotion of DNA repair activity and (iv) an enhancement of cell survival and dissemination machinery [97,98]. Even though previous investigations have found numerous molecules such as ATP-binding cassette (ABC) transporter proteins, nucleotide excision repair (NER) gene ERCC1, TP53, Aurora kinases and epithelium-mesenchymal transition (EMT) markers contributed to the resistance of treatment, mainly for chemotherapy, in HNSCC cells [98], successful reports to facilitate treatment sensitivity by targeting these resistance-associated factors are scarce. In addition, as most studies only describe the association between resistance and gene expression, it still remains unknown what the upstream inducers are for MDR gene alterations thereby leading to an acquired treatment resistance in HNSCCs.

Many lines of evidence indicate that THM compounds are promising to reduce multidrug resistance in different cancers. For example, ginsenoside Rh2 could inhibit P-glycoprotein (P-gp) activity, thereby suppressing multidrug resistance in breast cancer cells [99], whereas ginsenoside Rg3 could also ameliorate CDDP resistance via a downregulation of B7-H1 levels and resume T-cell cytotoxicity in human nonsmall cell lung cancer (NSCLC) cells [100]. Emodin, an anthraquinone derivative isolated from many plants including *Rheum palmatum*, *Polygonum cuspidatum*, *Polygonum multiflorum*, and *Cassia obtusifolia* was also reported to effectively facilitate cisplatin-induced cytotoxicity through multidrug resistance associated protein 1 (MRP1) down-regulation in human bladder cancer T24 and J82 cells [101]. Moreover, both in vitro and in vivo evidence implied that the combination of gambogic acid (GA), major compounds derived from gambogethe resin exuded from *garcinia hanburyi* and *garcinia Morella*, with doxorubicin, synergistically reduces cell viability in platinum-resistant human ovarian cancer SKOV3 cells and SKOV3-bearing xenografic tumors [102].

As for HNSCCs cells, a number of studies have showed that THM compounds could reverse multidrug resistance. An early study showed that Berberine, the major constituent of *Coptis Chinese*, modulated multidrug resistance gene, pgp-179 and sensitized paclitaxel-induced cytotoxicity [103]. Furthermore, artesunate could selectively inhibit cell growth through iron-dependent and Nrf2-mediated ferroptosis in CDDP resistant HN9 cells [104]. Targeting Nrf2 could also be applicable to reduce CDDP resistance by wogonin, a natural flavonoid found in root extract of *Scutellaria baicalensis* in HNSCC cells [105]. Other studies found that (-)-gossypol, a natural product isolated from cotton seeds and roots, could sensitize CDDP resistance in HNSCC cells, through regulations of tumor suppressor p53 status and apoptosis-related protein BCL-2 and BCL-xL [106,107]. At the cellular level, emerging evidence supports a concept that the tumor is derived from a distinct subset of cells with characteristics of self-renewal and differentiation capacity named cancer-initiating cells (CICs) or cancer stem cells (CSCs) [108]. As CICs/CSCs exhibit a quiescent slow-turnover phenotype, they are likely resistant to the conventional therapies which are often targeting highly proliferative cancer cells [109,110]. Following this concept, it was found that EGCG could attenuate stemness thereby enhancing CDDP chemosensitivity via the Notch signaling pathway, both in vitro and in vivo [111]. Other studies showing THM compounds displaying antiresistance impacts were also reported. For example, *Galium verum* aqueous extract profoundly suppressed cell invasion in Taxol-resistant human laryngeal carcinoma [55]. Danshen extract significantly inhibited proliferation of etoposide- and Taxol-resistant human oral cancer cells [112]. Finally, Celastrol triggered cell apoptosis in vincristine-resistant human tongue cancer cells via modulation of JNK1/2 signaling pathway [113]. Collectively, THM compounds could reduce drug resistance experimentally, both in vitro and in vivo, in HNSCC cells (Table 3).

### 2.4. Effects of THM Extracts for Radiotherapy-Induced Xerostomia/Oral Mucositis

HNSCC patients are often managed with preventive or therapeutic radiotherapy, which could result in frequent salivary gland damage and oral mucositis [114,115]. Salivary gland dysfunction could lead to reduced saliva secretion, which in turn gives rise to symptoms of xerostomia (dry mouth). Xerostomia affects patients’ quality of life by various pathophysiological conditions including oral discomfort, altered taste, difficulty of talking, swallowing and chewing as well as increased risk of dental diseases [116]. Oral lubricants, artificial saliva or saliva substitutes as well as pharmacologic treatment (e.g., pilocarpine) for xerostomia have been found to be temporally effective to improve xerostomia symptoms. However, only transient relief and reported adverse effects (of pilocarpine) limit their use [117,118]. Following the concept of integrative medicine, the guideline of using THM compounds as part of the management recommendations for xerostomia patients has been developed [119,120]. One open-label parallel study aiming to determine the effectiveness of a 4-week usage of an herbal compound containing Malva sylvestris and Alcea digitata powder compared to artificial saliva, showed a significant difference between two groups in relieving xerostomia symptoms [121]. Nevertheless, a study that systematically reviewed the outcomes of treatments with THM and without THM for over 900 HNSCC patients with radiotherapy-induced xerostomia in 14 different random controlled trails concluded that THM is not capable of significantly lessening xerostomia and related complications [122]. In brief, THM compounds seem not convincingly applicable to treat radiation-induced xerostomia.

Oral mucositis is also a radiotherapy-related pathological condition often found in HNSCC patients. Oral mucositis is characterized by inflammation or ulceration of the oral mucosa [115,123]. The most common features of oral mucositis include edema, erythema, ulcerations, bleeding, pain and xerostomia-related symptoms such as difficulty in swallowing, eating, drinking, talking and altered taste [124]. The concept of applying THM compounds for optimal treatment efficacy has been proposed and practiced in HNSCC patients with oral mucositis after radiotherapy or chemotherapy [125]. Mechanistically, THM compounds could help improve oral mucositis via several cues including anti-inflammation, immunomodulatory, antitoxic and antiseptic effects [124]. As most THM compounds exhibit anti-inflammatory activity, the underlying mechanism is thought to be down-regulation of proinflammatory cytokines (e.g., IL-1, IL-6, IL-8, and TNF-α) and up-regulation of anti-inflammatory cytokines (e.g., IL-4, IL-13, IL-10, and TGF-β) [125]. Recently, several systematic review articles, which compiled evidence-based studies for further determining the usefulness of THM agents in the management of oral mucositis induced by chemotherapy or radiotherapy in HNSCC patients, were reported. Taken together, it seems that most trials showed promising results stating that use of THM compounds could ease oral mucositis in HNSCC subjects, both in a preventive and therapeutic manner [126,127]. Further investigations are still required to determine the underlying molecular basis of antioral mucositis effects of each THM compound and cross-talk between various herbs since the knowledge of multiherbal combination therapy is not yet revealed.

## 3. Conclusions

THM compounds could exert anti-HNSCC properties by targeting different cellular cues. Further investigations are still required to comprehensively determine the underlying molecular basis of individual anti-HNSCC THM compounds. The cross-talk between various herbs in multiherbal combination therapy schemes is also of great interest to explore. In addition, the advantage of using THM in modulating treatment efficacy by FDA-approved immunotherapy monoclonal antibodies targeting immune checkpoint molecules Programmed cell death protein 1 (PD-1) and its receptor Programmed death-ligand 1 (PD-L1) (e.g., nivolumab and pembrolizumab) in HNSCC patients with relapse or metastatic CDDP-resistant tumors should also be closely followed. Lastly, better understanding of availability, administration route, effective doses and patient compliance upon THM application would be also of importance to feasibly translate THM compounds in aiding quality of life of HNSCC patients.

## Figures and Tables

**Table 1:** Regulatory Effects of Traditional Herbal Medicine (THM) compounds for Head and Neck Squamous Cell carcinoma (HNSCC) Cell Growth/Survival.

Herbal Compounds	Cell Types	Cellular/Molecular Changes
Curcumin	NT8e (HNSCC cells) Combination of 5-FU or doxorubicin (DOX)	↓cyclins (D1, E2, B1, and A2) and CDK2 ↑p21 levels → cell cycle growth arrest at the G1/S phase ↓EGFR-ERK1/2 signaling molecules → cell proliferation↓ ↓Bcl-2 ↑Bax, caspase-3, and PARP →apoptosis↑ [12]
	Copper supplementation of curcumin in several oral cancer cells	↑anti-tumor growth [35]
	OE33/OE19 (Human esophageal adenocarcinoma)	↑T cell-induced cytotoxicity [36]
Epigallocatechin gallate (EGCG)	KB (p53 wild-type human oral cancer) FaDu (Human hypopharynx squamous cell carcinoma)	↓mRNA and transcriptional activity of β-catenin KB cells ↑ubiquitination and proteasomal degradation of β-catenin ↑apoptosis [37]
	SCC-4 (Human tongue squamous carcinoma)	↑BAD, BAK, FAS, IGF1R, WNT11, and ZEB1 genes ↓CASP8, MYC, and TP53 ↑cell death via apoptosis and autophagy [32]
Berberine	KYSE70 (Human esophageal squamous carcinoma) SKGT43 (Human esophageal adenocarcinoma cell)	↓phosphorylation of Akt ↑AMP-activated protein kinase phosphorylation ↑apoptosis [22]
Artemisinin (Dihydroartemisinin)	5-8F/CNE-1/CNE-2/CNE-2Z (Human nasopharyngeal carcinoma cells)	↑ferroptosis and apoptosis [38]
	CAL-27 (Human head and neck squamous cell carcinoma)	↑LC3B-II level→autophagy↑ [33]
	Combined DHA and PDT treatment in human esophageal cancer cell line Eca109 cells ^##^ tumor	↓HIF-1α and VEGF ↓cell/tumor growth in vitro and in vivo ^##^ [39]
Ursolic acid (UA)	Ca922 (Human oral squamous cell carcinoma)	↓Akt/mTOR/NF-κB signaling ↓ERK/p38 [23]
Triptolide	CNE (Human nasopharyngeal carcinoma)	↓NF-κB p65 phosphorylation →anti-tumor [40]
	KYSE180 (well differentiated) Eca109 (well differentiated) KYSE150 (poor differentiated) (Human esophageal squamous carcinoma)	↑ caspases activity →cycle arrest at the G1/S phase and apoptosis↑ ↓ p53 and MAPK/ERK signaling pathway regulation →regulates cell apoptosis [17]
Cucurbitacins	SAS (Human tongue squamous carcinoma)	↑caspases activity → apoptosis [18]
Tanshinones	SCC-9 (Human tongue squamous carcinoma)	↑Beclin-1/Atg7/Atg12-Atg5 pathway ↓PI3K/Akt/mTOR pathway →autophagy↑ [24]
Oridonin	KYSE-30/KYSE-150/EC9706 (Human esophageal squamous carcinoma)	↓cyclin B1, CDK2 and Bcl-2 ↑p53, p21, Bax, cleaved caspase-3, -8, and -9 ↓PI3K/Akt/mTOR and Ras/Raf signaling pathway in vivo ^##^ ↑cell cycle arrest [13]
	UM1 and SCC-25 (Human oral squamous cell carcinoma)	↑the ratio of Bax/Bcl-2 ↑the cleavage of caspase-3, caspase-9 and PARP-1 ↓proliferation and clonal formation ↑apoptosis ↓cyclin B1 ↑cyclin D1, cyclin D3, P21, p-CDK1 and cyclin A2 →G2/M phase arrest [29]
Chrysophanol	FaDu (Human hypopharynx squamous cell carcinoma) SAS (Human tongue squamous carcinoma)	↑cleaved caspase-3 ↑ROS ↑apoptosis ↓G1 phase arrest [31]
Shikonin	5-8F (Human nasopharyngeal carcinoma)	↓plasma membrane integrity →electron-lucent cytoplasm and intact nuclear membrane → necroptosis↑ ↑RIPK1, RIPK3, and MLKL ↓tumor growth in the 5-8F xenograft mouse model ^##^ [41]
Artesunate	Eca109/Ec9706 (Human esophageal squamous carcinoma)	↓BCL-2 and CDC25A ↑Bax and caspase-3 → apoptosis and cell cycle arrest↑ ^##^ In vivo: dose-dependent tumor regression [19]
Wogonin	NPC-TW076/NPC-TW039 (Human nasopharyngeal carcinoma)	↓mTOR/P70S6K pathway →autophagy↑ ↓Raf/ERK and PI3K/Akt pathway ↑apoptosis [25]
β-Elemene	YD-38 (Human gingival squamous cell carcinoma) in vitro and in vivo ^##^	↓p-STAT3, p-JAK2, and Bcl-2 ↑Bax and caspase-3 ↑proliferative inhibition and apoptosis [30]
Demethoxycurcumin	SCC-9, HSC3 (Human tongue squamous carcinoma)	↓cIAP1/XIAP ↑HO-1 ↑cleaved caspase-3, -8, -9 ↓p38 →G2/M phase arrest ↑apoptosis [26]
Moscatilin	FaDu (Human hypopharynx squamous cell carcinoma)	↑cleaved caspase-3, -7, -8, -9 ↑cleaved PARP ↓JNK activity ↑apoptosis [27]
Cepharanthine (CEP)	CNE-1/CNE-2 (Human nasopharyngeal carcinoma)	↓NF-κB ↑apoptosis [14]
	HSC2, HSC3 and HSC4 (Human oral squamous cell carcinoma) in vitro and in vivo ^##^	↓DNA double-strand break (DSB) repair after radiation ↑caspase-3 → apoptosis↑ [42]

^##^: In vivo study. Abbreviations: DHA: Dihydroartemisinin; PDT: Photodynamic therapy; ERK: extracellular signal-regulated kinase; MAPK: mitogen-activated protein kinase; PARP: Poly(ADP-ribose) polymerase; Nrf2: Nuclear factor erythroid 2-related factor 2; cIAP1/XIAP: cellular IAP 1/X-chromosome-linked IAP; HO-1: heme oxygenase-1; JNK = c-Jun N-terminal kinase; ROS: Reactive oxygen species.

**Table 2:** Regulatory Impacts of THM compounds for HNSCC Cell Mobility/Metastasis and Angiogenesis.

Herbal Compounds	Cell Types		Cellular/Molecular Changes
*Andrographis paniculata*	EC-109 (Human esophageal squamous carcinoma)		↓WNT/BMP pathway ↓ErbB and MAPK pathway [45] ↓TM4SF3, HER2, CXCR4, NFκB, MMP-2 and MMP-9 [46]
	^##^ Intraperitoneal EC-109 xenograft mouse		↓liver and lung metastases [46] ↓tumor weight and tumor nodule number [47]
*Andrographispaniculate extract*/isoandrographolide	EC-109/KYSE-520 (Human esophageal squamous carcinoma) HMEC-1 (Human microvascular endothelial cell)		↓anoikis resistance, TM4SF3 [47]
*Eclipta* *prostrata*	HSC-3/SCC-9 (Human tongue squamous carcinoma) TW2.6 (Human buccal carcinoma)		↓MMP-2 (probably via ERK1/2 signaling pathways) [48]
*Rubus* *Idaeus*	SCC-9/SAS (Human tongue squamous carcinoma)		↓MMP-2 (probably via ERK1/2 signaling pathways) [49,50]
	HONE-1, NPC-39 and NPC-BM (Human nasopharyngeal carcinoma)		
*Selaginella* *tamariscina*	HSC-3 (Human tongue squamous carcinoma)		↓MMP-2 and MMP-9 ↑TIMP-1 and TIMP-2 (probably via Akt pathway) [51]
*Duchesnea* *indica*	SCC-9/SCC-14 (Human tongue squamous carcinoma) TW2.6 (Human buccal carcinoma)		↓MMP-2 (probably via MEK/ERK1/2 signaling pathways) [52]
*Leucaena* *leucocephala*	SCC-9/SAS (Human tongue squamous carcinoma)		↓MMP-2 (probably via ERK1/2 and p38 signaling pathways) [53]
*Physalis* *angulata*	HSC-3 (Human tongue squamous carcinoma)		↓VEGF, MMP-2, MMP-9 and u-PA ↑TIMP-1, TIMP-2, PAI-1 and PAI-2
	HUVECs	In vitro	↓MMP-2
		^##^ In vivo (CAM assay)	↓Induced neovascularization [54]
*Galium* *verum*	Hep-2/HLaC79 (Taxol sensitive/resistant human laryngeal carcinoma)		↓MMP-2 [55]
*Rheum palmatum* L.	SCC-9/SAS (Human tongue squamous carcinoma)		↓MMP-2 (probably via ERK1/2 signaling pathways) [56]
*Rheum palmatum* L./Emodin, aloe-emodin and rhein	SCC-4 (Human tongue squamous carcinoma)		↓MMP-2 and u-PA [57]
*Rheum palmatum* L./Chrysophanol	FaDu (Human hypopharynx squamous cell carcinoma) SAS (Human tongue squamous carcinoma)		↓EMT (↑E-cadherin, ↓vimentin) [31]
*Rhizomacoptidis*/Berberine	SCC-4 (Human tongue squamous carcinoma)		↓MMP-2, MMP-9, and u-PA [58]
*Gynostemmapentaphyllum* Makino/Gypenosides	SAS (Human tongue squamous carcinoma)		↓MMP-2, MMP-7, and MMP-9 [59]
Myrtaceae pollen and *Eucalyptus* honey/Tricetin	SCC-9/HSC-3 (Human tongue squamous carcinoma) OECM-1 (Human oral epidermal carcinoma)		↓MMP-9 (probably via p38/JNK1/2 pathway) [60]
*Pinussylvestris*/pinosylvin	SAS/SCC-9/HSC-3 (Human tongue squamous carcinoma)		↓MMP-2 ↑TIMP-2 (probably via ERK1/2 signaling pathways) [61]
*Salvia miltiorrhiza* (Danshen)/salvianolic acid A	SCC-9/SCC-25 (Human tongue squamous carcinoma)		↓MMP-2 (probably via c-Raf/MEK/ERK pathway) [62]
Quercetin (found in onion)	SAS (Human tongue squamous carcinoma)		↓MMP-2 and MMP-9 [63]
Phenethyl isothiocyanate	SAS (Human tongue squamous carcinoma)		↓p-EGFR, MMP-2 and MMP-9 ↑TIMP-1 and TIMP-2 (probably via PI3K/AKT, NF-κB, and MAPK pathway) [64]
Gallic Acid	NPC-BM1 (Human nasopharyngeal carcinoma)		↓MMP-1 ↑TIMP-1 (mediated by ↓AP-1, ETS-1, p-p38, c-fos and c-jun) [65]
Evodiamine	HONE1 (poorly differentiated) and CNE1 (well differentiated) (Human nasopharyngeal carcinoma)		↓MMP-2 (probably via ↓NF-κB p65, p-ERK1/2) [66]
Ursolic acid (UA)	Ca922 (Human oral squamous cell carcinoma)		↓angiogenesis ↓migration/invasion by blocking MMP-2 secretion [23]
Nobiletin	HONE-1 (Human nasopharyngeal carcinoma) NPC-BM (Human nasopharyngeal carcinoma derived from bone marrow metastatic lesion)		↓MMP-2 ↑TIMP-2 (probably via ERK1/2, NF-kB, and AP-1 pathway) [67]
	^##^ HONE-1 injected s.c. into the right flank of BALB/c nude mouse		↓lung metastasis (↓NF–κB) [67]
Resveratrol	SCC-9 (Human tongue squamous carcinoma)		↓MMP-9 (probably via JNK1/2 and ERK1/2 pathways) [68]
Triptolide	CNE (Human nasopharyngeal carcinoma)		anti-angiogenesis [40]
	KYSE180 (well differentiated) Eca109 (well differentiated) KYSE150 (poor differentiated) (Human esophageal squamous carcinoma)		differentially regulates metastasis [17,69]
Pinostilbene hydrate (methylated derivative of resveratrol)	SAS/SCC-9/HSC-3 (Human tongue squamous carcinoma)		↓MMP-2 (probably via p38/ERK1/2 pathway) [70]
Epigallocatechin gallate (EGCG)	TW01 (Human keratinizing squamous cell carcinoma) TW06 (Human undifferentiated nasopharyngeal carcinoma		↓stem cell genes (Oct4 and Klf4) ↓EMT related protein (↓Snail, Vimentin/↑E-Cadherin) [71]
	TW01 (EBV-negative)/NA (EBV-positive), (Human nasopharyngeal carcinoma) NP460hTert (Human immortalized human nasopharyngeal cell)		↓MMP-2 and MMP-9 (mediated by ↓ERK, AP-1 and Sp1 [72]
	CNE2 and C666-1 (Human nasopharyngeal carcinoma)		↓EMT (via NF-κB p65 inactivation) [73]
	^##^ CNE2-SC xenograft nude mouse (combined with cisplatin treatment)		↓N-cadherin, vimentin, Bmi-1, Twist1, and NF-κB p65 ↑E-cadherin [73]
	CAL-27 (HNSCC cell) (treated alone or combined with Gefitinib)		↓MMP-2, p-EGFR ↑TIMP-2 (probably via MAPK pathway) [74]
Qigesan (*fufang*)	TE1, TE13 and Eca109 (Human esophageal cancer)		↑Cx26 and Cx43 [75]
Fuzheng Yiliu granules	^&&^ Randomized clinical treatment (treatment group = 30, control group = 33) (combined with radiotherapy)		↑RBC-C3bRR ↓RBC-ICRR and CD44v6 [76]
Aidi injection	EC9706/KYSE70 (Human esophageal squamous carcinoma)		↓VEGF-A, cadherin-2 and vimentin ↑cadherin-1 [77]
	^##^ EC9706 cells inoculated into the peritoneal cavity of BALB/c NU mouse		↓vimentin and VEGF-A ↑cadherin-1 [77]

^##^: In vivo study. ^&&^: Clinical study. Abbreviations: MMP = metalloproteinase; u-PA = urokinase-type plasminogen activator; Cx = connexin; VEGF = vascular endothelial growth factor; TIMP = tissue inhibitor of matrix metalloproteinase; ERK = extracellular signal-regulated kinase; MAPK = mitogen-activated protein kinase; JNK = c-Jun N-terminal kinase (JNK); BMP = bone morphogenetic protein; TKI = tyrosine kinase inhibitor; EGFR = epidermal growth factor receptor; HIF-1α = Hypoxia inducible factor 1, alpha subunit; THBS2 = Thrombospondin 2; TGF = Transforming Growth Factor; AP-1 = activator protein-1; NF-κB = nuclear factor-κB; PAI = plasminogen activator inhibitors; HUVECs = human umbilical vein endothelial cells; CAM = chick chorioallantoic membrane; EBV = Epstein-Barr virus; ETS-1 = proto-oncogene 1.

**Table 3:** Regulatory Impacts of THM compounds for HNSCC Cell Drug Resistance.

Herbal Compounds	Cell Types	Cellular/Molecular Changes
Berberine	OC2 (Human oral cancer cells)	↑Paclitaxel induced cytotoxicity ↓multidrug resistant gene pgp-170 [103]
Artesunate	Eca109/Ec9706 (Human esophageal squamous carcinoma)	↓mitochondrial membrane potential [19]
Wogonin	AMC-HN4R/AMC-HN9R (CDDP resistant HNSCC cells)	↓cell number ↓Nrf2 and glutathione S-transferase P → ROS accumulation↑ →cell death pathways involving PUMA and PARP↑ [105]
EGCG	K3, K4 and K5 (Cancer stem cells isolated from HNSCC patients)	↓sphere forming capacity ↓Oct4, Sox2, Nanog and CD44 ↓ABCC2 and ABCG2 → ↑CDDP mediated chemosensitivity ↓xenografic tumor formation and induced apoptosis^##^ [111]
(-)-Gossypol	UM-SCC-5/UM-SCC-10B (CDDP sensitive/resistant HNSCC cells)	→induced more cell apoptosis in CDDP resistant cells via regulation of Bcl-2 and Bcl-xL as well as p53 status [106,107]
*Galiumverum* aqueous extract	Hep-2/HLaC79 (Paclitaxel sensitive/resistant human laryngeal carcinoma)	↑MDR and p-gp protein expression in resistant cells →more profoundly inhibited 3D spheroid mediated invasion [55]
Danshen extract	KB-7D (etoposide resistant) KB-tax (taxol resistant) (Human oral cancer)	→significantly inhibited the proliferation of drug-resistant cells [112]
Celastrol	SAS/SASV16 (vincristine-resistant human tongue squamous carcinoma)	→G2/M cell cycle arrest ↑caspases-3, -8, -9 and PARP activity → apoptosis →modulating BCL-2 and JNK1/2 signaling activity [113]
Cepharanthine (CEP)	Eca109 (CDDP sensitive/resistant human esophageal squamous cell carcinoma) In vitro and in vivo ^##^	↓P-gp →sensitivity of cell lines resistant to cisplatin↑ [14]

^##^: In vivo study. Abbreviations: P-gp: P-glycoprotein; MDR: Multidrug resistance; ABCG2: ATP-binding cassette super –family G member 2; JNK = c-Jun N-terminal kinase (JNK).
